# Migraine Disability Improvement during Treatment with Galcanezumab in Patients with Chronic and High Frequency Episodic Migraine

**DOI:** 10.3390/neurolint15010017

**Published:** 2023-02-13

**Authors:** Francesca Schiano di Cola, Marco Bolchini, Salvatore Caratozzolo, Giulia Ceccardi, Matteo Cortinovis, Paolo Liberini, Renata Rao, Alessandro Padovani

**Affiliations:** 1 Neurology Unit, Department of Clinical and Experimental Sciences, University of Brescia, 25122 Brescia, Italy; 2 Neurology Unit, Department of Neurological and Vision Sciences, ASST Spedali Civili, 25122 Brescia, Italy

**Keywords:** migraine, CGRP, disability, burden, MIDAS, HIT-6, galcanezumab

## Abstract

Background: The aim of the present study was to assess the migraine outcome, in particular migraine disability, in chronic (CM) and high frequency episodic migraine (HFEM) patients in treatment with galcanezumab. Methods: The present study was conducted at the Headache Centre of Spedali Civili of Brescia. Patients were treated with galcanezumab 120 mg monthly. Clinical and demographical information were collected at the baseline (T0). Data about outcome, analgesics consumption and disability (MIDAS and HIT-6 scores) were collected quarterly. Results: Fifty-four consecutive patients were enrolled. Thirty-seven patients had a diagnosis of CM, 17 of HFEM. During treatment, patients reported a significant reduction in terms of mean headache/migraine days (*p* < 0.001), the attacks’ pain intensity (*p* = 0.001) and monthly consumed analgesics (*p* < 0.001). The MIDAS and HIT-6 scores also documented a significant improvement (*p* < 0.001). At the baseline, all patients documented a severe degree of disability (MIDAS score ≥ 21). Following six months of treatment, only 29.2% of patients still documented a MIDAS score ≥ 21, with one third of patients documenting little or no disability. A > 50% MIDAS reduction, compared to baseline, was observed in up to 94.6% of patients, following the first three months of treatment. A similar outcome was found for HIT-6 scores. A significant positive correlation was found between headache days and MIDAS at T3 and T6 (T6 > T3), but not baseline. Discussion: Monthly prophylactic treatment with galcanezumab was found to be effective in both CM and HFEM, especially in reducing migraine burden and disability.

## 1. Introduction

Migraine is classified as a primary headache disorder according to the International Headache Society (IHS) classification (International Classification Headache Disorders, ICHD-3) [1]. Along with tension-type headache, it is one of the most commonly observed neurological disorders in the general population. It is estimated that migraine affects 14.4% of the overall world population, with a predominant distribution among females, compared to males (18.9% vs. 9.8%) [2]. Most severe forms of migraine are high-frequency episodic migraine (HFEM), characterized by a frequency of 8 to 14 migraine days per month, and chronic migraine (CM), defined as a frequency of at least 15 monthly headache days (MHDs) with at least 8 with migraine characteristics, for 3 months [1]. These forms of migraine account for the high burden of this neurological disease, with an estimated 45.1 million years lived with disability (YLDs) [3], making migraine the second most disabling disease among all neurological disorders, and the leading cause of disability when considering women less than age 50 [4]. The impact and burden of migraine on the individual patient involves multiple settings, including occupational, social, familial, and personal [5].

Migraine burden occurs both during and between attacks (also referred to as ictal and interictal burden) [1]. Ictal burden is correlated to the actual symptoms of migraine attacks, with limitations, mainly, on personal functioning, whereas interictal burden has an impact on the ability to plan events and activities, mainly correlated to the fear of possible new attacks [6,7]. In addition to the actual pain phase, migraine can be accompanied by a plethora of other manifestations, as visual disturbances (e.g., visual snow), osmophobia, allodynia (i.e., when a normal non-noxious stimulus from touch of the skin is perceived as painful or a cause of discomfort), pain on movement, motion sickness, vestibular dysfunctions, cognitive symptoms, and cranial autonomic symptoms. Patients with migraine may experience these symptoms even in the interictal phase, although generally less severely [6,7,8,9]. The headache phase can be preceded by up to 72 h by a prodrome phase, the “premonitory phase”, that includes symptoms like fatigue, difficulty in concentrating, neck stiffness, photo- and phono- phobia, nausea, blurred vision, yawning, and pallor, among other phenomena [3]. Many patients can predict a migraine attack from the onset of the preliminary symptoms with a fair degree of accuracy, even hours before the onset of pain [10]. A postictal phase can then follow the headache pain [3] and last up to 24 h. Reported symptoms include fatigue, sleepiness, difficulty concentrating, and mild residual head discomfort [11]. The postdrome phase can be highly disabling for patients. A study on CM patients reported up to 63% of patients being “very/extremely limited” in their daily activities during the postdromal phase [12]. 

When considering interitcal burden, an Italian study found a significant association between executive disturbances and the duration and intensity of migraine attacks, together with mild executive dysfunction during the interictal phase, in patients with a diagnosis of migraine without aura [13]. A few studies have also examined objective findings regarding the interictal migraine disability. One study using functional brain MRI documented an altered global sensory processing during the interictal state in patients with migraine without aura [14]. Recently, a very interesting study has been published, in which the authors followed for 21 days one episodic patient via proton magnetic resonance spectroscopy (1H-MRS) and functional resting state MRI daily [15]. The main outcome of this study is that changes in N-acetyl aspartate (NAA) levels were associated with altered mitochondrial metabolism and thus abnormal energy metabolism. This phenomenon was observed in the occipital lobe and the basal ganglia, and it might reflect abnormal energy metabolism and may increase the perceptivity of migraine patients to excitatory migraine triggers.

Considering the high burden of this disease, assessment of migraine-related disability is essential for a proper treatment to be adopted. Several tools are available to assess migraine burden, with the Migraine Disability Assessment Scale (MIDAS) being one of the most-widely used. It is a self-administered five-item questionnaire assessing the number of days of wholly lost or highly decreased activity in school, work, household and family, and social or leisure activities due to migraine [16]. It has been validated in different languages worldwide, as well as in Italian [17]. Scores range from 0 to a maximum of 270 and are categorized into four severity groups: little-to-no (0–5 points), mild (6–10), moderate (11–20) and severe (>20). The MIDAS score has a moderately high test–retest reliability [5], with previous studies documenting a correlation between the MIDAS summary score and the equivalent diary score around 0.63 [18].

Another commonly used tool to assess migraine-related disability is the short form Headache Impact Test 6 (HIT-6) [19], a six-item questionnaire assessing activity limitations in different domains, pain severity, fatigue, frustration, and difficulty in concentration [20].

It was developed for use in a general headache population [17]. Each item is rated using five response categories (“Never, Rarely, Sometimes, Very often, or Always”); each category of which is associated with a numerical value (6, 8, 10, 11, and 13, respectively), resulting in a range of possible total summed scores of 36–78. Scores above 60 are associated to a severe migraine disability. Assessment of migraine-related disability is particularly important during preventive treatment as it aids the clinician in assessing the overall treatment efficacy. Indeed, it has been identified by the American Headache Society as one of three clinically relevant tools for assessing the benefit of preventive treatment in migraine patients [21]. Thus, it has also been used as a measure of impact in clinical trials investigating headache and migraine treatments [22,23,24,25,26,27]. The HIT-6 has also been validated in the CM trial population [28] as a specific patient reported outcome in this class of patients. The appropriateness and efficacy in being a patient-centered scale of migraine disability was all confirmed by a recent review that focused on the HIT-6 questionnaire [29].

Among preventive treatments, monoclonal antibodies targeting the Calcitonin gene-related peptide (CGRP) system play a major role in the treatment of high frequency and chronic migraine.

CGRP is a 37-amino acid peptide primarily localized to C and Aδ sensory fibers. The trigemino-vascular system is involved in the regulation of the cranial vasculature and is a key element in the transmission of pain [30]. In a study by Edvinsson and Goadsby [31], it was shown that only CGRP is released in significant amounts during migraine and cluster headache attacks. Further studies supported these results by demonstrating increased levels of CGRP in the serum, cerebrospinal fluid, and saliva of migraine patients [32]. Moreover, the elevated levels of CGRP normalize following effective triptan treatment of the migraine attack. Systemic administration of CGRP in migraine patients is sufficient to trigger a migraine-like attack phenotypically similar to the subject’s usual migraine attack [33]. CGRP is also localized in nonneuronal tissues, of which less is known at present. Given its highly potent vasodilator effect, it stands as a protective mechanism in the cardiovascular system and wound healing [30].

Among monoclonal antibodies targeting the CGRP ligand, galcanezumab is a highly specific and potent humanized antibody [34]. Galcanezumab has a linear pharmacokinetic, with generally dose-proportional increases when administered as a single subcutaneous dose [35,36]. The time to maximum concentration was around 5 days and steady-state concentrations for the 120 mg maintenance dose were achieved by month 1 when a loading dose of 240 mg was administered in patients with migraine [36,37]. Its half-life is about 27 days [38].

Galcanezumab is recognized as an effective treatment in both episodic [39,40] and chronic migraine [41], significantly reducing the number of monthly migraine days compared to a placebo in randomized controlled trials (RCTs), respectively, EVOLVE-1, EVOLVE-2 and REGAIN. In EVOLVE-1, galcanezumab 120 and 240 mg (once-monthly) were associated with significantly greater overall reductions in monthly migraine headache days than a placebo during the double-blind treatment phase (change from baseline −4.7 and −4.6 vs. −2.8; *p* < 0.001 for both comparisons) [40]. The onset of effect was month 1 in both treatment groups and a benefit was seen from week 1 in a subsequent post-hoc analysis [nuov17]. Similar results were observed in the EVOLVE-2 study [40]. Moreover, there was a significantly greater reduction in the number of days with acute medication use in the treatment group compared to the placebo (change −3.7 and −3.6 vs. −1.9; *p* < 0.001) [18]. Compared to the placebo group, a significantly greater proportion of patients in the treatment group achieved a ≥50% clinical response. Regarding migraine disability, evaluated using the MSQ Role Function-Restrictive domain scores and the PGI-S scores, mean improvements from the baseline during treatment were significantly greater with galcanezumab 120 and 240 mg than with the placebo [40].

Similar results were observed in CM patients in the REGAIN study [41].

Evidence from real-life clinical practice supports these findings [42,43,44,45], although it is still limited. In particular, rapid response, consistent conversion from chronic to episodic migraine and significant reduction of medication overuse have all been reported [46,47]. Long term efficacy in the real-world setting has also been discussed [48].

In particular, limited data exists regarding anti-CGRP monoclonal antibodies’ efficacy beyond the reduction of migraine days, i.e., pain intensity and migraine disability.

The aim of the present study was to assess the migraine outcome, in terms of headache frequency, pain severity, analgesics consumption and migraine disability (MIDAS and HIT-6 scores), during treatment with galcanezumab in patients with episodic and chronic migraine. Moreover, we aimed to assess whether the correlation between MIDAS/HIT6 scores and MHDs, in our cohort, (1) was consistent with previous data from the literature and (2) was modified by migraine improvement and if so by what extent.

## 2. Methods

### 2.1. Standard Protocol Approvals and Patient Consents

This study received approval from the ethics standards committee on human experimentation (local ethics committee of the ASST—Azienda Socio Sanitaria Territoriale, Spedali Civili Hospital, Brescia: NP 3949, approved 10 August 2020). Full written informed consent was required for all participants.

### 2.2. Study Design and Participants

The present work is an monocenter observational retrospective cohort study conducted at the Headache Centre—Neurology Clinic at the ASST Spedali Civili Hospital of Brescia from November 2020 to January 2022. The study included all adult patients with a diagnosis of HFEM or CM in prophylactic treatment with galcanezumab with an available 6 months’ follow-up. Inclusion criteria were the following: documented history of migraine for at least 12 months, headache diary compilation in the 3 months prior to galcanezumab’s introduction and throughout the study period, ≥8 migraine days per month for at least 3 months, ≥3 previous prohylactic failures or contraindications or scares tolerance. The only accepted prophylactic failures, according to the Italian regulations, were anti-epileptic drugs (e.g., topiramate, valproic acid, lamotrigine, gabapentin), tricyclic antidepressants (e.g., amitryptiline, nortriptyline), beta blockers (e.g., propranolol, metaprolol, atenolol) and Onabotulinumtoxin A (for CM patients only). The exclusion criteria were a documented history of cerebrovascular disease and/or myocardiocal infarction, uncontrolled systemic hypertension, documented Raynaud disease (not the mere phenomenon), and severe constipation.

Patients were assessed at the baseline (T0) and following three (T3) and six (T6) months of treatment. Patients were treated with a galcanezumab subcutaneous injection with a first loading dose of 240 mg at T0 and then 120 mg monthly.

At the baseline, patients’ data regarding their migraine history (e.g., disease duration, age at chronification) and features (e.g., associated symptoms, allodynia, pain localization), clinical and demographical information, previous and current acute and preventive migraine treatments, and concomitant medications were collected. Medication overuse was diagnosed based on the ICHD-3 [1]. Ictal allodynia was assessed qualitatively. Monthly headache and migraine days (respectively MHDs and MMDs), monthly analgesics consumption, and the attacks’ pain intensity (using the Numerical Rating Scale, NRS) were also collected at T0, T3 and T6. Patients were also asked to complete migraine disability questionnaires (HIT-6 and MIDAS) at the baseline and then at T3 and T6.

### 2.3. Outcome Measures

The objective of this analysis was to assess the clinical outcome of migraine patients in prophylaxis with galcanezumab, in terms of both migraine symptoms and migraine disability.

The primary endpoint was to assess headache frequency (MHDs, MMDs), pain intensity, analgesics consumption and migraine disability (MIDAS and HIT-6 scores) at T0, T3 and T6.

The following secondary endpoints were also evaluated: (1) the percentage of patients with a MIDAS score ≥50% reduction at T3 and T6 compared to baseline; (2) the correlation between MHDs/MMDs, MIDAS and HIT-6 scores at baseline (T0), and follow-up (T3 and T6).

### 2.4. Statistical Analysis

The Shapiro—Wilk test and Levene test were used to assess the normality of the distribution and the homogeneity of variance. Continuous variables were described as mean and standard deviation or median and interquartile range as appropriate, categorical variables were expressed as frequencies and percentages.

A one-way repeated measures ANOVA was conducted to test whether there were statistically significant differences in MMDs/MHDs, pain intensity, analgesics consumption and migraine disability (MIDAS and HIT-6 scores) from baseline to T3 and T6.

A Spearman correlation coefficient between MHDs/MMDs, HIT-6 and MIDAS scores was assessed in all patients at the baseline, T3 and T6.

Statistical significance was set at *p* < 0.05. Data analyses were carried out with SPSS software (version 22.0; Armonk, NY, USA).

## 3. Results

Fifty-four consecutive patients were enrolled, of whom 50 (92.5%) were female. No patient dropped out due to a lack of efficacy or adverse events. At the baseline, 37 (68.5%) of patients were affected by CM and 17 (31.5%) by HFEM. Forty patients (74.1%) also presented with MO. On average, patients had failed 4.4 (SD 2.1) previous preventive treatments. Among CM patients, 17 (45.9%) had previously failed Onabotulinumtoxin A treatment. Mean disease duration was 30.1 years (SD 10.6). Clinical and demographical variables are presented in Table 1. Consistent with their diagnosis, patients affected by CM documented significantly more MHDs and MMDs; they also documented a higher analgesics consumption, with a higher frequency of MO compared to episodic patients. Measures of disability were also significantly higher than episodic patients, in particular the MIDAS scale (respectively, 111.1 vs. 65; *p* = 0.01). Of notice, pain intensity during migraine attacks did not differ between chronic and episodic patients (respectively, 7.7 vs. 7.6; *p* = 0.7).

**Figure 1:**
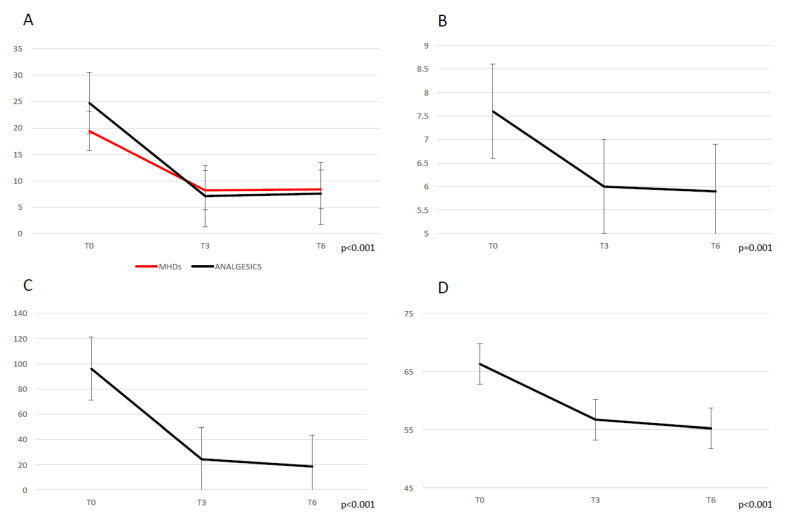
Migraine outcome during treatment with galcanezumab, following 3 (T3) and 6 (T6) months. (**A**) monthly headache days (MHDs) and analgesics consumption. (**B**) mean migraine attacks’ pain severity (NRS score). (**C**) MIDAS score. (**D**) HIT-6 score.

Patients reported a significant reduction in terms of MHDs (*p* < 0.001) from the baseline (19.4 ± 7.5) to T3 (8.2 ± 6.5) and T6 (8.4 ± 7.05); MMDs (*p* < 0.001) from the baseline (11.2 ± 7.3) to T3 (2.8 ± 3.02) and T6 (3.7 ± 5.9); the attacks’ pain intensity measured using the NRS (*p* = 0.001) from the baseline (7.6 ± 1.1) to T3 (6.02 ± 1.7) and T6 (5.9 ± 1.9); monthly consumed analgesics (*p* < 0.001) from the baseline (24.7 ± 14.7) to T3 (7.1 ± 6.9) and T6 (7.6 ± 8.9). Similarly, a significant reduction (*p* < 0.001) in both MIDAS and HIT-6 scores was found from the baseline (respectively 96.1 ± 70.8 and 66.3 ± 5.1) to T3 (24.3 ± 24.7 and 56.7 ± 9.2) and T6 (18.6 ± 19.6 and 55.2 ± 11.2). All data are shown in Figure 1.

**Figure 2:**
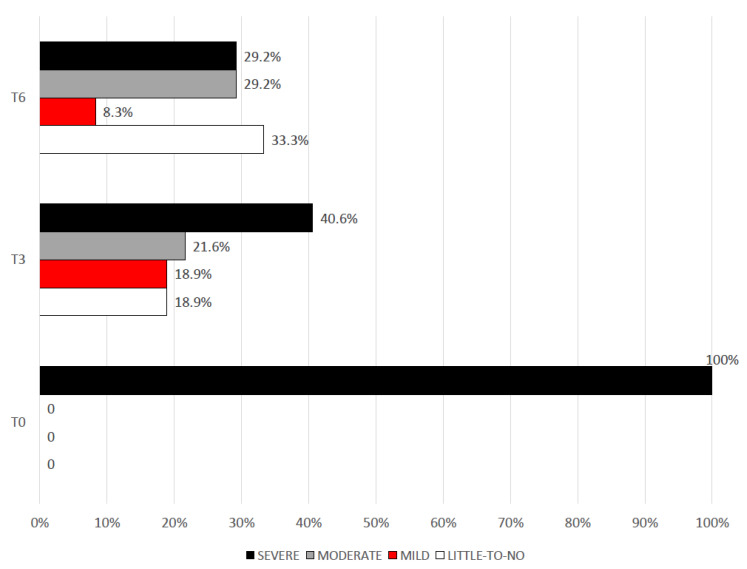
Degrees of migraine disability (MIDAS score) from the baseline and following 3 (T3) and 6 (T6) months of treatment.

MIDAS scores were then categorized according to the degree of disability in “little to no disability” (score 0–5), “mild” (score 6–10), “moderate” (score 11–20), “severe” (score ≥ 21). At the baseline, all patients fell into the severe disability group. At T3, 40.5% of patients were still in the severe disability group. At T6, less than a third of patients (29.2%) still had a MIDAS score ≥ 21, with 33.3% of patients documenting little or no disability (see Figure 2).

Overall, at T3 and T6, respectively, up to 94.6 and 93.8% of patients documented a >50% reduction in their MIDAS score, compared to baseline. On average, patients lost up to −74.3% (SD, 18.5) of their initial MIDAS score at T3, which went up to −80.6% (SD, 23.8) at T6.

Similarly, the HIT-6 scores were categorized as above or below 60. At the baseline 95.6% of patients documented a HIT-6 score above 60. This percentage decreased to 24.3 and 29.2% at T3 a T6, respectively.

**Figure 3:**
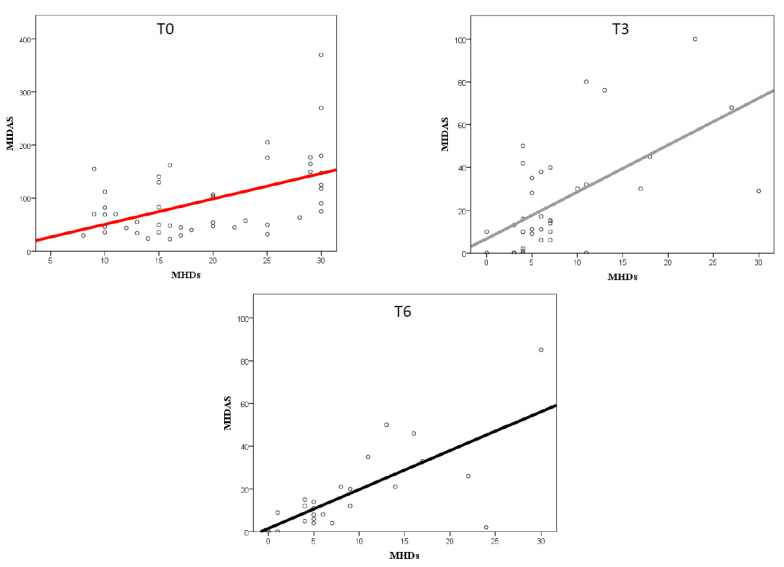
Correlation between MIDAS score and migraine frequency (MHDs) at the baseline (T0) and following 3 (T3) and 6 (T6) months of treatment.

At the baseline, a significant but moderate correlation (*p* < 0.0001) between MHDs and MIDAS scores (r = 0.58) was found, which became stronger at T3 (r = 0.61) and T6 (r = 0.71), as presented in Figure 3. The correlation between MHDs and HIT-6 scores was significant only at T6 (r = 0.59; *p* = 0.002).

Similarly, the correlation between MIDAS and HIT-6 scores grew stronger from baseline (r = 0.36; *p* = 0.01) to T3 (r = 0.58; *p* < 0.0001) to T6 (r = 0.63; *p* = 0.002).

## 4. Discussion

The present data confirmed galcanezumab to be effective in patients with both HFEM and CM in improving headache frequency, pain intensity, analgesics consumptions and migraine disability (MIDAS and HIT-6 scores). We confirm the data reported in the RCTs EVOLVE 1 and 2 and REGAIN [39,40,41], but also of previous real-life clinical studies. In particular, we confirm preliminary reports regarding the more extensive improvement in real-life studies (−8 days HFEM, −13 days CM) [42,43] compared to RCTs. Indeed, on average our patients lost up to 11 days from the baseline to T6.

Currently, more and more attention is being paid to migraine disability, i.e., assessing the benefits of prophylactic treatment beyond headache days. Our findings documented a significant improvement not only on migraine frequency but also on pain intensity, analgesics consumption and scales of migraine disability.

The MIDAS scores were divided into categorical items (little to no disability, mild, moderate, and severe) and while at the baseline all patients reported severe disability, both at T3 and at T6 a significant reduction of disability was observed, with less than one third of patients still reporting severe disability at T6 and more than 90% of patients achieving a >50% reduction of MIDAS score. Similarly, the HIT-6 scores at the baseline were categorized as above or below 60. During treatment, the percentage of patients still documenting a score above 60 was less than 30%.

The MIDAS and HIT-6 questionnaires focus on different aspects of migraine, the former on migraine disability in general and the latter on migraine disability related to the actual migraine episodes [29,49]. It is noteworthy that both domains improved significantly during treatment. A further analysis was conducted to assess the relationship between headache days and migraine-related disability. Interestingly, at baseline the correlation between headache frequency and MIDAS scores was found to be moderate, possibly meaning that the number of headache/migraine days did not directly correlate and could not explain per se the high degree of disability observed. During treatment, the degree of the correlation between headache frequency and the MIDAS score increased and became stronger. This finding suggests that disability in migraine patients, especially in patients with more disabling forms of migraine (high frequency and chronic), should be considered as a complex phenomenon, not only related to the sole number of headache days, but most importantly related to a wider spectrum of manifestations (impacting personal functioning, social and familial aspects of life, and the psychological burden of the disease and its consequences). Thus, the stronger correlation observed between the MHDs and MIDAS score following galcanezumab’s introduction might suggest a treatment benefit not only in terms of headache frequency but also on other more subtle aspects of the disease. As a consequence, while at baseline disability measured with MIDAS seems not strongly correlated with headache days (as other more complex factors contribute to this disability), during treatment the correlation becomes stronger, and the observed disability seems mostly due to the remaining headache days observed. Similar results were observed between the MHDs and the HIT-6 score, with a significant correlation at T6 but not at T3 or baseline. Similar results were found in a recent study addressing the correlation between disability measures and headache days in patients treated with galcanezumab [50,51].

We do acknowledge that the present study has some limitations. Firstly, the limited cohort, especially considering patients with episodic migraine. Given the current regulations regarding anti-CGRP prescription, episodic patients often fail to have all the legibility criteria, thus most patients in treatment with all monoclonal antibodies tend to have a CM diagnosis. This latter category tends to document higher degrees of disability and, of course, headache days. Secondly, we only assessed migraine disability using the MIDAS and HIT-6 scores. Further studies will be needed in order to evaluate the effect of CGRP monoclonal antibodies on the migraine ictal and interictal burden and quality of life, on larger cohorts. In particular, it would be of interest to evaluate scales of interictal burden like the Migraine Interictal Burden Scale (MIBS-4) questionnaire [52,53] or composite measures like the total pain burden (frequency of migraine headache days in a month, duration of migraine headache on a given day, and maximum severity of migraine headache on a given day) [54]. To date only one study has evaluated the total pain burden in a real-life setting [45], with most studies coming from post-hoc analyses of clinical trials [54,55]. Moreover, future research should focus on the identification of response predictors, not only in terms of migraine frequency but, indeed, migraine disability.

## 5. Conclusions

In conclusion, the present data confirm galcanezumab’s efficacy in migraine prevention in a real-life scenario in patients with episodic and chronic migraine. A significant improvement in both migraine frequency (both headache and migraine days) and migraine-related disability, as measured with the MIDAS and HIT-6 scores, was observed, as well as a significant reduction in monthly analgesics consumption and the mean attacks’ pain intensity. Interestingly, migraine disability and frequency documented only a moderate correlation at baseline, as multiple factors could be involved in this complex relationship. This correlation became stronger during treatment, suggesting that preventive treatment might be beneficial not only in improving on migraine frequency, but also on the elements contributing to migraine burden.

## Figures and Tables

**Table 1:** Baseline clinical and demographical characteristics.

	Total (*n* = 54)	HFEM (*n* = 17)	CM (*n* = 37)	*p*
Age (years) mean, SD	46.2 (9.5)	47.2 (11.3)	45.7 (8.6)	0.1 ^#^
Female, *n* (%)	50 (92.5)	15 (88.2)	35 (93.5)	0.9 ^§^
Medication overuse, *n* (%)	40 (74.1)	7 (41.1)	34 (91.8)	0.0001 ^§^
Disease duration (years) mean, SD	30.1 (10.6)	33.4 (10.6)	28.4 (10.3)	0.8 ^#^
Allodynia, *n* (%)	29 (53.7)	9 (52.9)	20 (54)	1.0 ^§^
Previous failed prophylaxis mean, SD	4.4 (2.1)	3.7 (2.3)	4.8 (2)	0.2 ^#^
MHDs T0 mean, SD	19.4 (7.5)	12.4 (5.3)	22.8 (5.9)	0.04 ^#^
MMDs T0 Mean, SD	11.2 (7.3)	8.6 (3.1)	12.1 (8.1)	0.02 ^#^
Monthly total analgesics consumption T0 mean, SD	24.7 (14.7)	13.2 (7.5)	30.3 (28.4)	0.02 ^#^
- NSAIDs	6.7 (12.1)	4.2 (8.9)	7.8 (13.5)	0.3 ^#^
- Triptans	11.5 (8.9)	8.9 (5.3)	12.8 (8.1)	0.03 ^#^
- Combination	6.5 (25.8)	0 (0)	9.6 (30.2)	0.02 ^#^
Pain intensity-NRS T0 mean, SD	7.6 (1.1)	7.6 (0.9)	7.7 (1.2)	0.7 ^#^
MIDAS score T0 mean, SD	96.1 (70.8)	65 (38)	111.1 (78.3)	0.01 ^#^
HIT-6 score T0 mean, SD	66.3 (5.1)	65.3 (3.7)	66.8 (5.6)	0.3 ^#^

*n*: number; SD: standard deviation; MHDs: monthly headache days; MMD: monthly migraine days; T0: baseline; MIDAS: Migraine Disability Assessment Score; HIT-6: Head Impact Test; NSAIDs: non-steroidal anti-inflammatory drugs; NRS: numerical rating scale; ^#^: Independent *t*-test; ^§^: Chi square.

## Data Availability

The data presented in this study are available on request from the corresponding author. The data are not publicly available due to ethical restrictions.

## References

[1] Headache Classification Committee of the International Headache Society (2018). The International Classification of Headache Disorders. Cephalalgia.

[2] Burch R.C., Buse D.C., Lipton R.B. (2019). Migraine: Epidemiology, Burden, and Comorbidity. Neurol. Clin..

[3] Feigin V.L., Nichols E., Alam T., Bannick M.S., Beghi E., Blake N., Culpepper W.J., Dorsey E.R., Elbaz A., Ellenbogen R.G. (2019). Global, regional, and national burden of neurological disorders, 1990–2016: A systematic analysis for the Global Burden of Disease Study 2016. Lancet Neurol..

[4] Stovner L.J., Nichols E., Steiner T.J., Abd-Allah F., Abdelalim A., Al-Raddadi R.M., Ansha M.G., Barac A., Bensenor I., Doan L.P. (2018). Global, regional, and national burden of migraine and tension-type headache, 1990–2016: A systematic analysis for the Global Burden of Disease Study 2016. Lancet Neurol..

[5] Buse D.C., Scher A.I., Dodick D.W., Reed M.L., Fanning K.M., Adams A.M., Lipton R.B. (2016). Impact of Migraine on the Family: Perspectives of People with Migraine and Their Spouse/Domestic Partner in the CaMEO Study. Mayo Clin. Proc..

[6] Ashkenazi A., Mushtaq A., Yang I., Oshinsky M. (2009). Ictal and Interictal Phonophobia in Migraine—A Quantitative Controlled Study. Cephalalgia.

[7] Lovati C., D’Amico D., Bertora P., Rosa S.D.S.R.F., Suardelli M., Mailland E., Mariani C., Bussone G. (2007). Acute and Interictal Allodynia in Patients With Different Headache Forms: An Italian Pilot Study. Headache.

[8] Gossrau G., Frost M., Klimova A., Koch T., Sabatowski R., Mignot C., Haehner A. (2022). Interictal osmophobia is associated with longer migraine disease duration. J. Headache Pain.

[9] Main A., Dowson A., Gross M. (1997). Photophobia and phonophobia in migraineurs between attacks. Headache.

[10] Giffin N., Ruggiero L., Lipton R., Silberstein S., Tvedskov J.F., Olesen J., Altman J., Goadsby P., Macrae A. (2003). Premonitory symptoms in migraine: An electronic diary study. Neurology.

[11] Blau J.N. (1982). Resolution of migraine attacks: Sleep and the recovery phase. J. Neurol. Neurosurg. Psychiatry.

[12] Giffin N.J., Lipton R.B., Silberstein S.D., Olesen J., Goadsby P.J. (2016). The migraine postdrome: An electronic diary study. Neurology.

[13] Camarda C., Monastero R., Pipia C., Recca D., Camarda R. (2007). Interictal Executive Dysfunction in Migraineurs Without Aura: Relationship with Duration and Intensity of Attacks. Cephalalgia.

[14] Meylakh N., Henderson L.A. (2022). Exploring alterations in sensory pathways in migraine. J. Headache Pain.

[15] Filippi V., Steiger R., Beliveau V., Frank F., Kaltseis K., Gizewski E.R., Broessner G. (2022). Investigating the Migraine Cycle over 21 Consecutive Days Using Proton Magnetic Resonance Spectroscopy and Resting-State fMRI: A Pilot Study. Brain Sci..

[16] Stewart W.F., Lipton R.B., Kolodner K., Liberman J., Sawyer J. (1999). Reliability of the Migraine Disability Assessment Score in A Population-Based Sample of Headache Sufferers. Cephalalgia.

[17] D’Amico D., Mosconi P., Genco S., Usai S., Prudenzano A., Grazzi L., Leone M., Puca F.M., Bussone G. (2001). The Migraine Disability Assessment (MIDAS) Questionnaire: Translation and Reliability of the Italian Version. Cephalalgia.

[18] Stewart W.F., Lipton R.B., Kolodner K.B., Sawyer J., Lee C., Liberman J.N. (2000). Validity of the Migraine Disability Assessment (MIDAS) score in comparison to a diary-based measure in a population sample of migraine sufferers. Pain.

[19] Kosinski M., Bayliss M.S., Bjorner J.B., Ware J.E., Garber W.H., Batenhorst A., Cady R., Dahlöf C.G.H., Dowson A., Tepper S. (2003). A six-item short-form survey for measuring headache impact: The HIT-6™. Qual. Life Res..

[20] Bayliss M.S., Batenhorst A.S. (2002). The HIT-6™: A User’s Guide.

[21] Society A.H. (2019). The American Headache Society Position Statement on Integrating New Migraine Treatments into Clinical Practice. Headache.

[22] Aurora S.K., Dodick D.W., Diener H., DeGryse R.E., Turkel C.C., Lipton R.B., Silberstein S.D. (2014). OnabotulinumtoxinA for chronic migraine: Efficacy, safety, and tolerability in patients who received all five treatment cycles in the PREEMPT clinical program. Acta Neurol. Scand..

[23] Lipton R.B., Rosen N.L., Ailani J., DeGryse R.E., Gillard P.J., Varon S.F. (2016). OnabotulinumtoxinA improves quality of life and reduces impact of chronic migraine over one year of treatment: Pooled results from the PREEMPT randomized clinical trial program. Cephalalgia.

[24] Matharu M., Halker R., Pozo-Rosich P., DeGryse R., Adams A.M., Aurora S.K. (2017). The impact of onabotulinumtoxinA on severe headache days: PREEMPT 56-week pooled analysis. J. Headache Pain.

[25] Blumenfeld A.M., Stark R.J., Freeman M.C., Orejudos A., Adams A.M. (2018). Long-term study of the efficacy and safety of OnabotulinumtoxinA for the prevention of chronic migraine: COMPEL study. J. Headache Pain.

[26] Ashina M., Dodick D., Goadsby P.J., Reuter U., Silberstein S., Zhang F., Gage J.R., Cheng S., Mikol D.D., Lenz R.A. (2017). Erenumab (AMG 334) in episodic migraine: Interim analysis of an ongoing open-label study. Neurology.

[27] Gaul C., Diener H.-C., Danesch U., on behalf of the Migravent^®^ Study Group (2015). Improvement of migraine symptoms with a proprietary supplement containing riboflavin, magnesium and Q10: A randomized, placebo-controlled, double-blind, multicenter trial. J. Headache Pain.

[28] Rendas-Baum R., Yang M., Varon S.F., Bloudek L.M., DeGryse R.E., Kosinski M. (2014). Validation of the Headache Impact Test (HIT-6) in patients with chronic migraine. Health Qual. Life Outcomes.

[29] Houts C., Wirth R., McGinley J.S., Gwaltney C., Kassel E., Snapinn S., Cady R. (2020). Content Validity of HIT-6 as a Measure of Headache Impact in People with Migraine: A Narrative Review. Headache.

[30] Russell F.A., King R., Smillie S.-J., Kodji X., Brain S.D., Pressly J.D., Soni H., Jiang S., Wei J., Liu R. (2014). Calcitonin Gene-Related Peptide: Physiology and Pathophysiology. Physiol. Rev..

[31] Goadsby P.J., Edvinsson L. (1993). The trigeminovascular system and migraine: Studies characterizing cerebrovascular and neuropeptide changes seen in humans and cats. Ann. Neurol..

[32] Edvinsson L., Ekman R., Goadsby P.J. (2010). Measure-ment of vasoactive neuropeptides in biologicalmaterials: Problems and pitfalls from 30 years ofexperience and novel future approaches. Cephalalgia.

[33] Hansen J.M., Hauge A.W., Olesen J., Ashina M. (2010). Calcitonin gene-related peptide triggers migraine-like attacks in patients with migraine with aura. Cephalalgia.

[34] Benschop R.J., Collins E.C., Darling R.J., Allan B., Leung D., Conner E., Nelson J., Gaynor B., Xu J., Wang X.-F. (2014). Development of a novel antibody to calcitonin gene-related peptide for the treatment of osteoarthritis-related pain. Osteoarthr. Cartil..

[35] Monteith D., Collins E.C., Vandermeulen C., Van Hecken A., Raddad E., Scherer J.C., Grayzel D., Schuetz T.J., De Hoon J. (2017). Safety, Tolerability, Pharmacokinetics, and Pharmacodynamics of the CGRP Binding Monoclonal Antibody LY2951742 (Galcanezumab) in Healthy Volunteers. Front. Pharmacol..

[36] Eli Lilly and Company (2018). EMGALITY (galcanezumab-gnlm) Injection, for Subcutaneous Use: US Prescribing Information. http://pi.lilly.com/.

[37] Kielbasa W., O’Brien L., Moser B., Quinlan T. (2018). Assessment of pharmacokinetics, target engagement and immunogenicity in patients with migraine administered galcanezumab, an anti-CGRP antibody [abstract no. IOR09]. Headache.

[38] Vermeersch S., Benschop R.J., Van Hecken A., Monteith D., Wroblewski V.J., Grayzel D., de Hoon J., Collins E.C. (2015). Translational Pharmacodynamics of Calcitonin Gene-Related Peptide Monoclonal Antibody LY2951742 in a Capsaicin-Induced Dermal Blood Flow Model. Experiment.

[39] Skljarevski V., Matharu M., Millen B.A., Ossipov M.H., Kim B.-K., Yang J.Y. (2018). Efficacy and safety of galcanezumab for the prevention of episodic migraine: Results of the EVOLVE-2 Phase 3 randomized controlled clinical trial. Cephalalgia.

[40] Stauffer V.L., Dodick D.W., Zhang Q., Carter J.N., Ailani J., Conley R.R. (2018). Evaluation of Galcanezumab for the Prevention of Episodic Migraine: The EVOLVE-1 Randomized Clinical Trial. JAMA Neurol..

[41] Detke H.C., Goadsby P.J., Wang S., Friedman D.I., Selzler K.J., Aurora S.K. (2018). Galcanezumab in chronic migraine: The randomized, double-blind, placebo-controlled REGAIN study. Neurology.

[42] Vernieri F., Altamura C., Brunelli N., Costa C.M., Aurilia C., Egeo G., Fofi L., Favoni V., Pierangeli G., Lovati C. (2021). Galcanezumab for the prevention of high frequency episodic and chronic migraine in real life in Italy: A multicenter prospective cohort study (the GARLIT study). J. Headache Pain.

[43] Vernieri F., Altamura C., Brunelli N., Costa C.M., Aurilia C., Egeo G., Fofi L., Favoni V., Lovati C., Bertuzzo D. (2022). Rapid response to galcanezumab and predictive factors in chronic migraine patients: A 3-month observational, longitudinal, cohort, multicenter, Italian real-life study. Eur. J. Neurol..

[44] Martin V., Samaan K.H., Aurora S., Pearlman E.M., Zhou C., Li X., Pallay R. (2020). Efficacy and Safety of Galcanezumab for the Preventive Treatment of Migraine: A Narrative Review. Adv. Ther..

[45] Silvestro M., Tessitore A., Orologio I., De Micco R., Tartaglione L., Trojsi F., Tedeschi G., Russo A. (2022). Galcanezumab effect on “whole pain burden” and multidimensional outcomes in migraine patients with previous unsuccessful treatments: A real-world experience. J. Headache Pain.

[46] Vernieri F., Altamura C., Aurilia C., Brunelli N., Egeo G., Fofi L., Costa C.M., Fallacara A., Favoni V., Pierangeli G. (2020). Effectiveness, safety, and tolerability of galcanezumab in a real-life setting in patients with migraine in Italy (the GARLIT study). Neurol. Sci..

[47] Altamura C., Brunelli N., Marcosano M., Aurilia C., Egeo G., Lovati C., Favoni V., Perrotta A., Maestrini I., Schiano Di Cola F. (2022). Conversion from chronic to episodic migraine in patients treated with galcanezumab in real life in Italy: The 12-month observational, longitudinal, cohort multicenter GARLIT experience. J. Neurol..

[48] Vernieri F., Brunelli N., Marcosano M., Aurilia C., Egeo G., Lovati C., Favoni V., Perrotta A., Maestrini I., Rao R. (2023). Maintenance of response and predictive factors of 1-year GalcanezumAb treatment in real-life migraine patients in Italy: The multicenter prospective cohort GARLIT study. Eur. J. Neurol..

[49] Stewart W.F., Lipton R.B., Dowson A.J., Sawyer J. (2001). Development and testing of the Migraine Disability Assessment (MIDAS) Questionnaire to assess headache-related disability. Neurology.

[50] Ford J.H., Stauffer V.L., McAllister P., Akkala S., Sexson M., Ayer D.W., Wang S. (2021). Functional impairment and disability among patients with migraine: Evaluation of galcanezumab in a long-term, open-label study. Qual. Life Res..

[51] Ayer D.W., Skljarevski V., Ford J.H., Nyhuis A.W., Lipton R.B., Aurora S.K. (2018). Measures of Functioning in Patients With Episodic Migraine: Findings From a Double-Blind, Randomized, Placebo-Controlled Phase 2b Trial With Galcanezumab. Headache.

[52] Buse D.C., Bigal M., Rupnow M., Reed M., Serrano D., Lipton R. (2007). Development and validation of the migraine Interictal burden scale (MIBS): A self-administered instrument for measuring the burden of migraine between attacks. Neurology.

[53] Buse D.C., Rupnow M.F.T., Lipton R.B. (2009). Assessing and managing all aspects of migraine: Migraine attacks, migraine-related functional impairment, common comorbidities, and quality of life. Mayo Clin. Proc..

[54] Ailani J., Andrews J.S., Rettiganti M., Nicholson R.A. (2020). Impact of galcanezumab on total pain burden: Findings from phase 3 randomized, double-blind, placebo-controlled studies in patients with episodic or chronic migraine (EVOLVE-1, EVOLVE-2, and REGAIN trials). J. Headache Pain.

[55] Ailani J., Andrews J.S., Tockhorn-Heidenreich A., Wenzel R., Rettiganti M. (2022). Effect of Galcanezumab on Total Pain Burden in Patients Who Had Previously Not Benefited from Migraine Preventive Medication (CONQUER Trial): A Post Hoc Analysis. Adv. Ther..

